# Effect of Informative Cesarean Delivery Operative Steps Video on Maternal Anxiety Level: A Randomized Controlled Trial

**DOI:** 10.12688/f1000research.147840.3

**Published:** 2024-12-23

**Authors:** Panicha Phetprapasri, Athita Chanthasenanont, Pichita Prasongvej, Winitra Nuallaong, Suphachai Chaitrakulthong, Densak Pongrojpaw

**Affiliations:** 1 Department of Obstetrics and Gynecology, Faculty of Medicine, Thammasat University, Khlong Luang, Pathum Thani, 12120, Thailand; 2 Department of Psychiatry, Faculty of Medicine, Thammasat University, Khlong Luang, Pathum Thani, 12120, Thailand; 3 Thammasat University Hospital, Thammasat University, Khlong Luang, Pathum Thani, 12120, Thailand

**Keywords:** Cesarean section, Anxiety, Informative video.

## Abstract

**Background:**

Cesarean section is the most common obstetric procedure performed. This can lead to maternal anxiety, which is a significant contributor to postpartum depression. This can adversely affect pregnant women both mentally and emotionally, negatively impacting their well-being and family bonding. The aim of this study was to evaluate the effect of the addition of an informative cesarean section operative steps video on the maternal anxiety score compared with standard pre-cesarean section care.

**Methods:**

This randomized controlled trial was conducted at Thammasat University Hospital, Thailand, between April and September 2023. Pregnant women who underwent their first cesarean section were allocated to two groups: intervention and control groups. Participants in the intervention group were required to watch a 5-minute informative video that elaborately described the process from pre-operative steps until post-operative care on the day scheduled for cesarean delivery. All participants received the same routine pre-operative and post-operative care. The State-Trait Anxiety Inventory (STAI) was used to measure both populations on two occasions: the day of scheduled cesarean delivery and postpartum day 1.

**Results:**

A total of 178 women were recruited. The demographic and obstetric characteristics were similar between the two groups. The pre-operative STAI scores of the intervention and control groups were 42.9 and 44.1 points, respectively, with no significant difference. However, the post-operative anxiety score showed a significant decline in the intervention group compared to that in the control group (p = 0.002). Moreover, most of the participants in the intervention group showed a low level of anxiety after the operation, while half of the control group remained at a moderate to high level score.

**Conclusions:**

The provision of an informative educational video before cesarean delivery is a powerful tool that significantly reduces cesarean operative anxiety and improves health outcomes.

Thai Clinical Trials Registry on the 28 March 2023 ( TCTR20230328001).

## Introduction

Cesarean section is the most common obstetric surgical procedure. The rate of cesarean deliveries increased globally by 19.4 percent in 2018. By 2030, the global cesarean section rate is projected to increase by 30 percent. This is equivalent to 38 million cesarean deliveries according to accumulated data. ^1^

In Thailand, the rate of cesarean delivery has shown a continuous upward trend, increasing from 25 percent to 30 percent over the last 15 years, and reaching 30 percent to 50 percent in 2023. ^2^^–^^4^ Cesarean sections have been associated with increased anxiety, heightened postpartum traumatic stress symptoms, and reduced well-being during pregnancy compared to vaginal delivery, ^5^ partically among women who did not initially planned for the procedure. ^6^^,^^7^ Maternal anxiety significantly contributes to postpartum depression. It is a common and serious issue that affects pregnant women, both mentally and emotionally. This can adversely affect one’s well-being and family bonding. Furthermore, cesarean delivery significantly increases the risk of postpartum depression. ^8^ Postpartum depression and anxiety can be influenced by various factors, such as maternal stress, anxiety during pregnancy, stressful events during or after childbirth, traumatic birth experiences, preterm birth, neonatal intensive care, low level of social support, previous history of depression, and breastfeeding problems. Screening for postpartum depression and anxiety during pregnancy using standardized assessment tools is recommended to efficiently identify and support at-risk women. ^9^

Previous research has indicated that anesthesia-specific informational videos can reduce anxiety levels and improve patient satisfaction during cesarean section procedures. ^10^ However, two separate reports have yielded contradictory findings. ^11^^,^^12^

The inconsistency in these results may be due to the fact that the videos in question focused solely on anesthesia-related information, without encompassing the comprehensive steps of pre-operative and post-operative care or the cesarean section procedure itself.

A study demonstrated that an informative video covering the entire cesarean process, from pre-operative preparations to post-operative recovery, was effective in reducing maternal anxiety both before and after surgery. ^13^ This finding suggests that a more holistic and well-structured video presentation may be more effective in alleviating anxiety.

Nonetheless, no research has been conducted to date in Southeast Asia including Thailand on the use of educational videos to reduce maternal anxiety in mothers preparing for cesarean sections. Such studies would ideally encompass detailed explanations of pre-operative and post-operative care, as well as the procedures that take place within the operating room.

The aim of this study was to investigate whether providing an informative cesarean section video before surgery would reduce maternal anxiety. Additionally, it aimed to assess the prevalence of anxiety in pregnant women undergoing cesarean delivery and evaluate the satisfaction level with the informative educational video.

## Methods

This randomized controlled trial was approved by the Human Ethics Committee of Thammasat University on the 21 March 2023 (MTU-EC-OB-1-010/66) and Thai Clinical Trials Registry on the 28 March 2023 ( TCTR20230328001). The study was conducted at the antenatal care clinic and postpartum wards at Thammasat University Hospital, Pathum Thani, Thailand. The participants were Thai singleton pregnant women aged between 18 and 45 years who were scheduled for cesarean delivery between April and September 2023. The exclusion criteria included previous cesarean delivery, pre-existing chronic illnesses before pregnancy, fetal complications during pregnancy, fetal congenital anomalies, severe early neonatal illnesses, pregnancy-related complications that could not be managed by medications, emergency surgery conditions, refusal to participate in this study, and pre-pregnancy mental disorders, such as a history of depression or anxiety, which have been shown to influence the risk of postnatal depression. Therefore, this study aims to minimize confounding factors associated with these conditions. ^7^

Patients who met the selection criteria underwent a thorough review before providing written informed consent, as approved by the ethic review committee, to participate in the study. All participants received standard prenatal care and delivery management, regardless of their involvement in the research. On the scheduled day for cesarean delivery, typically 1-2 weeks before the procedure, the research team provided comprehensive explanations about the study, including the randomization process. Participants were then randomly assigned to either the control or intervention group using block randomization. All participants received routine pre- and post-cesarean care, including an explanation of the surgical procedure and potential complications.

Participants in the intervention group were required to watch a 5-minute informative video, created specifically for this study, on the day of their scheduled cesarean delivery. The video contained no imagery or content that could induce anxiety and provided a detailed explanation of the pre-operative procedures, anesthesia, the cesarean delivery process, and post-operative care.

Before being implemented in the study, the video underwent a rigorous review by obstetricians at Thammasat University and was tested with patients to ensure clarity and accessibility.

Before randomization, the study included 178 participants. Sample size calculations, based on pilot data showing 20% post-cesarean anxiety, determined that 77 participants per group were needed to detect a reduction to 5% with the use of an informational video. An additional 10% was added to account for potential data loss, resulting in a total of 170 participants. Before assigning participants to either the intervention or control group, each participant completed a demographic questionnaire and the State-Trait Anxiety Inventory (STAI form Y-1). The reliability of the STAI was assessed over intervals ranging from one hour to 104 days, with the Trait-Anxiety scale showing reliability coefficients between 0.65 to 0.86. ^14^ The STAI form Y-1 questionnaire is on state anxiety and consists of 20 items evaluating four levels of intensity: not at all, sometimes, moderately, and very much. The questionnaire comprised 10 positively framed statements (Items 3, 4, 6, 7, 9, 12, 13, 14, 17, and 18), with scores ranging from 1 to 4. It also contained 10 negatively framed statements (Items 1, 2, 5, 8, 10, 11, 15, 16, 19, and 20) with scores ranging from 4 to 1. The total score ranged from 20 to 80 points, with a higher total score indicating a higher level of anxiety. The score interpretation was divided into three ranges of 20-39, 40-59 and 60-80 which can be interpreted as low, moderate, and high levels of anxiety, respectively. On postpartum day 1, all participants were reevaluated using the STAI from Y-1. If a research participant experiences a high level of anxiety before or after cesarean delivery, advice on meeting up with individual psychotherapy will be recommended, or a counseling team will offer assistance to help ease the participant’s anxiety. The intervention group was evaluated to measure the level of patient satisfaction with the informative videos on postpartum day 1. Statistical analysis was performed using Statistical Package for the Social Sciences version 26 (SPSS Inc., Chicago, USA). Continuous data were computed using an unpaired t-test and are presented as means with standard deviation. Categorical data were evaluated using the chi-square test and represented as numbers. Statistical significance was set than 0.05.

## Results

**Figure 1.**
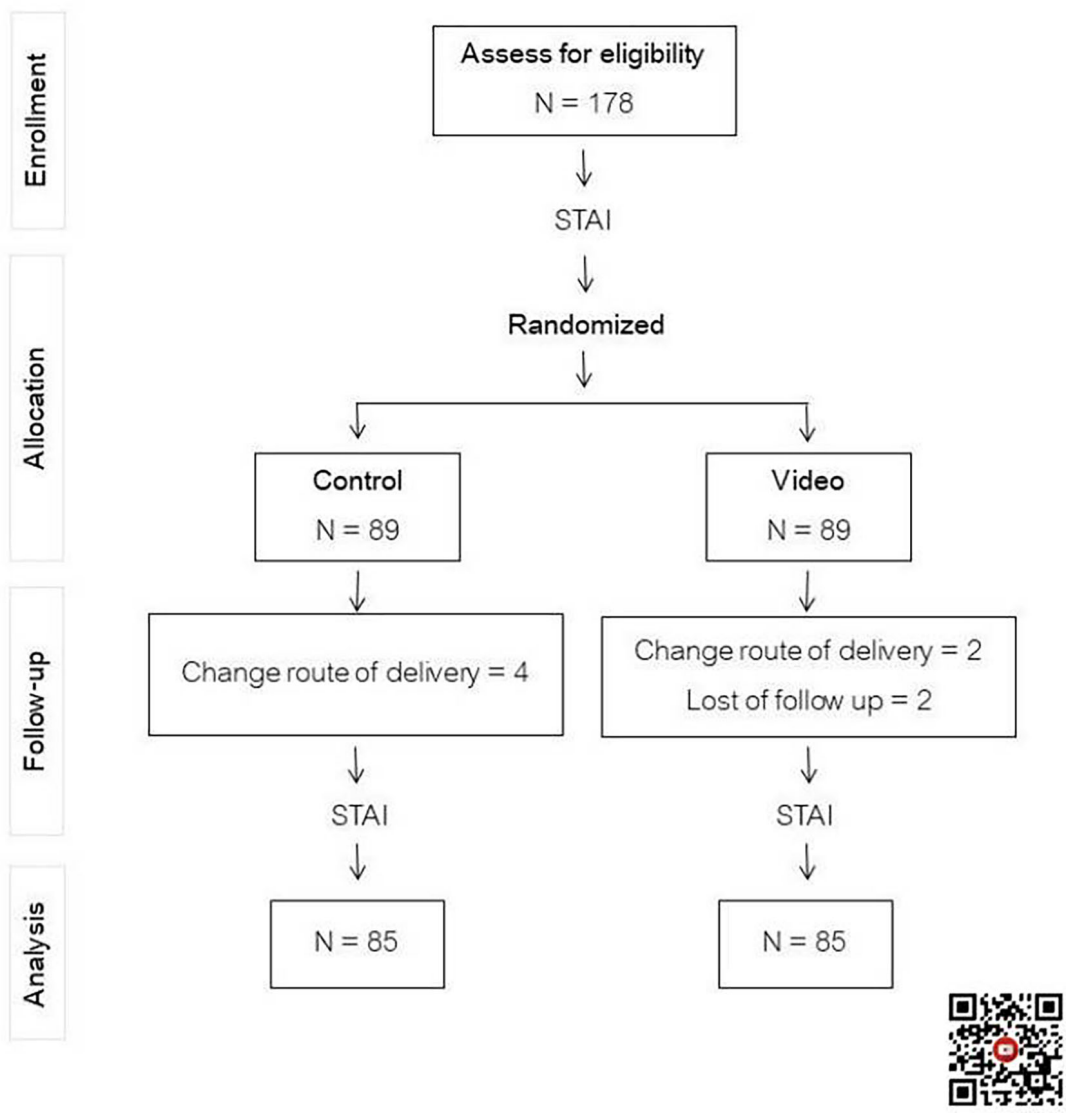
Flow chart of study. Control: standard pre-cesarean section operative care; Video: informative cesarean section operative steps video before cesarean section operation; STAI: State-Trait Anxiety Inventory. QR code for informative cesarean section operative steps video.

**Figure 2.**
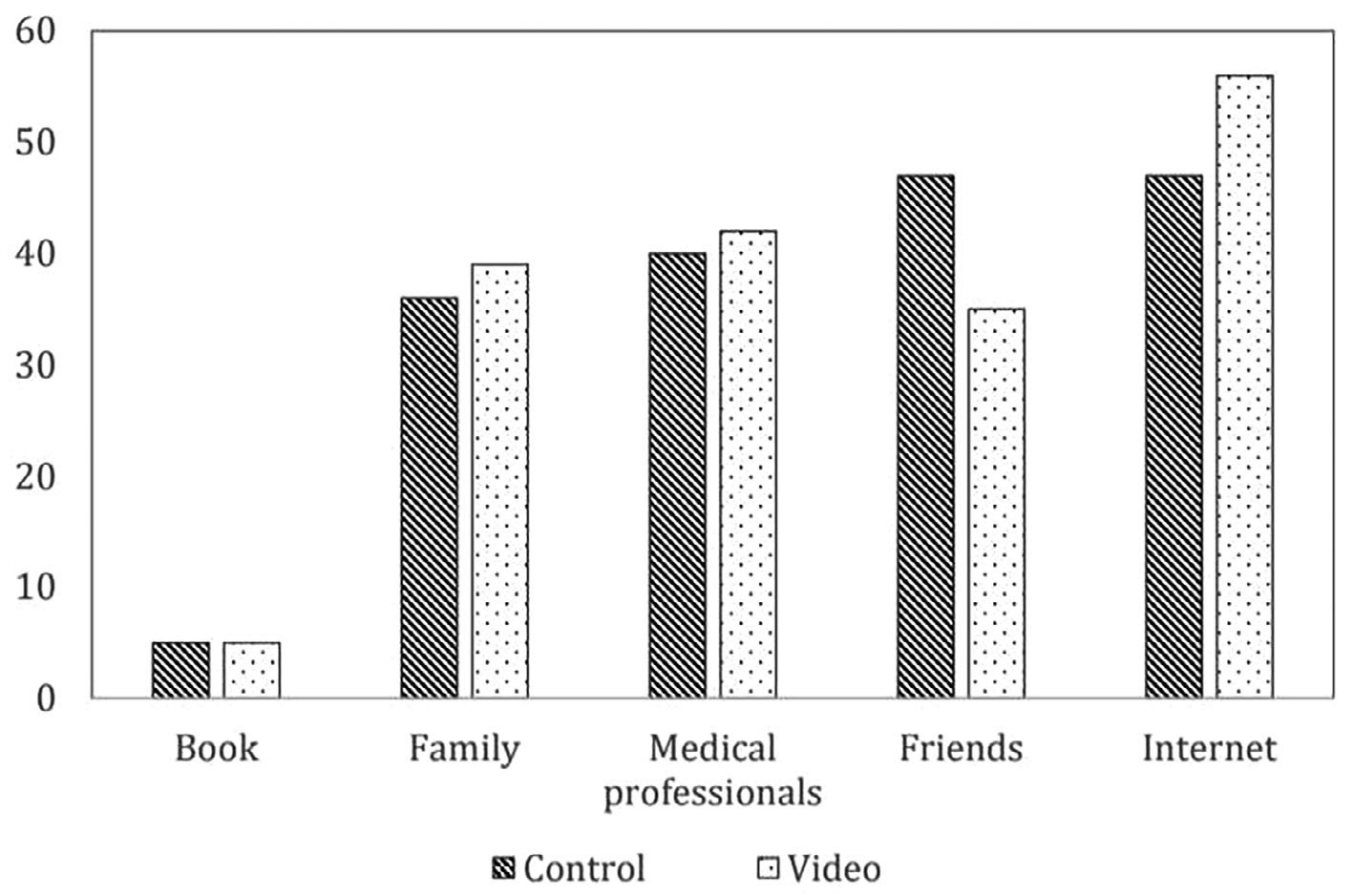
Information source of cesarean delivery before operation (not include intervention) (n=85 cases per group). Control: standard pre-cesarean operation care; Video: informative cesarean section steps video before operation; Book: The book that provides information on preparing for cesarean delivery; Family: A group of people who are related by blood, marriage or adoption such as parents; Medical professionals: person individuals trained and qualified to provide medical care such as doctors, nurses and other healthcare providers; Friends: person whom one has a bound of mutual affection who not a member of family; Internet: internet information providing guidance on preparing for cesarean delivery.

One hundred and seventy-eight participants scheduled for first-time cesarean delivery were recruited and equally randomized into two groups, with eight cases that dropped out, as shown in Figure 1. Maternal demographic and obstetric data between the two groups were statistically indistinguishable, as seen in Table 1. Most of the participants were primigravida, had an educational level of at least a bachelor’s degree, and did not have any underlying disease or previous abdominal surgery. No differences were found in the indications for cesarean section and incidence of postpartum hemorrhage in either group. There were no cases of serious post-operative complications such as re-exploration or neonatal intensive care unit admission. The information sources of cesarean delivery in both groups were similar, and most of the participants received information about cesarean delivery from the Internet and friends before joining the research, as shown in Figure 2.

**Table 1.** Demographic and obstetric characters of pregnant women who underwent first time cesarean section (n=85 cases per group).

	Control	Video	p-value
Age (years) *	32.07 ± 4.8	30.79 ± 5.3	0.100
BMI (kg/m ^2^) *	22.80 ± 4.5	23.56 ± 5.2	0.305
Nulliparity **	60 (70.6)	63 (74.1)	0.607
Education level **			0.123
≤ Secondary	19 (22.4)	28 (32.9)	
≥ Bachelor	66 (77.6)	57 (67.1)	
No underlying disease **	75 (88.2)	79 (92.9)	0.607
No history of abortion **	67 (78.8)	74 (87.1)	0.153
No history of abdominal surgery **	75 (88.2)	76 (89.4)	0.808
Indication **			0.583
Maternal request	52 (61.2)	60 (70.6)	
Malpresentation	14 (16.5)	14 (16.5)	
Obstructed labor	11 (12.9)	6 (7.1)	
Fetal macrosomia	4 (4.7)	2 (2.4)	
Placenta previa	4 (4.7)	3 (3.5)	
TR **	5 (5.9)	7 (8.2)	0.549
Weight of newborn *	3172.0 ± 406.6	3098.0 ± 478.0	0.278
Postpartum hemorrhage **	4 (4.7)	2 (2.4)	0.406

^*^ : mean ± standard deviation (SD).

^**^ : n (%); Control: standard pre-cesarean operation care; Video: informative cesarean section steps video before operation; BMI: body mass index; TR: Tubal sterilization operation.

Before cesarean delivery, the STAI scores of the intervention and control groups did not differ significantly. The prevalence of participants who had moderate to high levels of anxiety in this study was 71 percent, eight of whom had high STAI scores.

**Figure 3.**
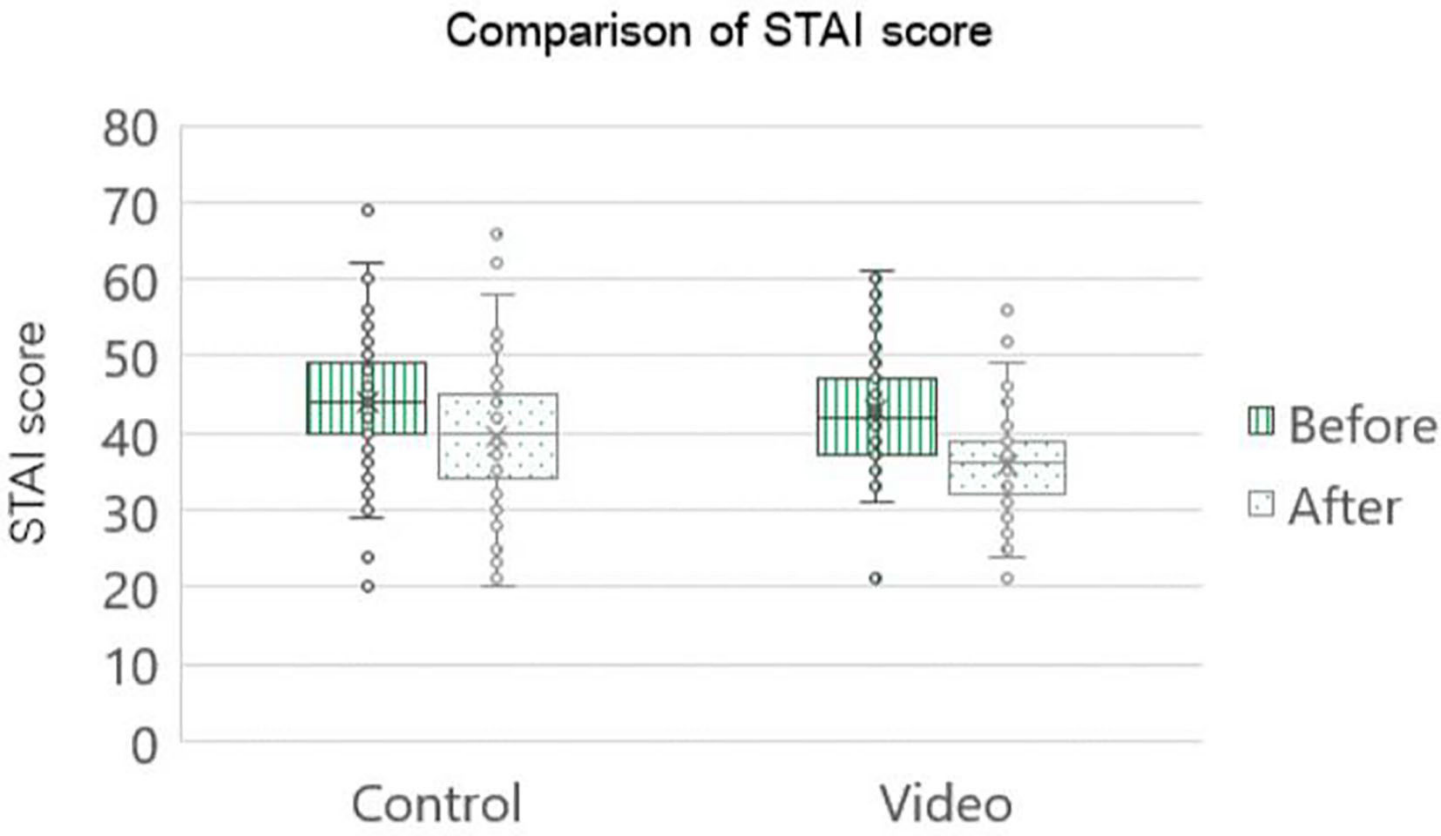
Comparison STAI score of pregnant women who underwent first time cesarean section (n=85 cases per group). STAI: The State-Trait Anxiety Inventory; Control: standard pre-cesarean operation care; Video: informative cesarean section steps video before operation; Before: anxiety score before cesarean section; After: anxiety score after cesarean section.

Post-operatively, the STAI score was statistically significant in both groups. However, upon juxtaposing the post-operative scores between the two groups, it became evident that the cohort exposed to the intervention group had a statistically noteworthy decrease in scores compared to the control group (decreasing mean STAI score were 7.0 and 4.4 in the intervention and control groups, respectively, p=0.002), as shown in Figure 3. Table 2 shows that the control group exhibited a pre-operative moderate-to-high anxiety level of 77 percent, which was reduced to 53 percent post-operatively. The intervention group showed that the proportion of patients with moderate-to-high anxiety levels decreased from 65 percent in the pre-operative period to 18% in the post-operative period.

**Table 2.** Comparison STAI score of pregnant women who underwent first time cesarean section (n=85 cases per group).

STAI levels	Control			Video		
	Before	After	p-value	Before	After	p-value
Low	19	40	<0.001	30	70	<0.001
Moderate–High	66	45		55	15	

STAI: State-Trait Anxiety Inventory (range 20-80); Control: standard pre-cesarean operation care; Video: informative cesarean section steps video before operation; before: anxiety score before cesarean section; after: anxiety score after cesarean section; Low: low level of anxiety (score 20-39); Moderate: moderate level of anxiety (score 40-59); High: high level of anxiety (score 60-80).

Seventy-eight percent of intervention patients transitioned from moderate to high anxiety pre-operatively to low anxiety post-operatively, doubling the control group's 34 percent improvement. Furthermore, two patients in the control group showed escalation from moderate to high anxiety levels, whereas in the intervention group, patients with high anxiety levels pre-operatively had reduced anxiety levels. Notably, all patients with pre- and post-operative high anxiety received psychological counseling. In the intervention group, almost all individuals reported the highest satisfaction scores for the informative cesarean section operative steps video, reaching approximately 87 percent.

According to the evaluation of factors affecting pre- and post-operative anxiety scores, pre-operative cesarean section anxiety was not affected. While two factors, watching the informative operative steps video and pre-operative anxiety scores, had a statistically significant impact on the post-operative anxiety score, the odds ratios were 0.17 and 8.23, respectively, as shown in Table 3. Watching the informative video had potentially reduced stress by 83%, and experiencing anxiety before surgery increased the risk of post-operative anxiety stress by 8.23 times.

**Table 3.** Logistic regression analysis to determine independents associated factors for pre- and post-operative STAI score.

	Adjusted OR	95 % CI	p-value
Pre-operative STAI score			
AMA	0.78	0.36–1.72	0.537
Nulliparity	1.34	0.44–4.10	0.614
Education level ≤ Secondary	1.62	0.67–3.94	0.289
Indication: maternal request	0.82	0.38–1.76	0.603
Post-operative STAI score			
AMA	0.60	0.24–1.49	0.269
Nulliparity	0.79	0.24–2.63	0.706
Education level ≤ Secondary	1.72	0.67–4.47	0.263
Indication: maternal request	1.85	0.79–4.34	0.157
Postpartum hemorrhage	4.01	0.49–32.91	0.196
Video	0.17	0.07–0.38	<0.001
Pre-operative anxiety scores	8.23	2.70–25.02	<0.001

STAI: State-Trait Anxiety Inventory; AMA: Advance maternal age ≥ 35 years; Video: Watching the informative operation steps video.

## Discussion

Maternal anxiety is prevalent throughout pregnancy, childbirth, and postpartum, with reported rates ranging from 15 percent to 36 percent. ^15^^–^^18^ Some studies suggest an increase in self-reported anxiety symptoms with advancing gestational age, peaking in the third trimester, and even exceeding postpartum levels. ^18^ Interestingly, our study found that in planned cesarean section, patients exhibited significantly higher anxiety levels (71 percent) compared to their counterparts without planned birth interventions during the third trimester. ^18^ This suggests a potential correlation between cesarean anticipation and heightened anxiety.

Cesarean section is the most common procedure in obstetrics and has been proven to save numerous lives of both mothers and infants during pregnancy complications. In some cases, parents may request this delivery method. There are various ways to access information regarding cesarean deliveries. However, despite its prevalence and readily available information, patients undergoing planned cesarean delivery still exhibit higher anxiety levels. Therefore, the ACOG strongly recommends assessing anxiety risks and conditions in pregnant and postpartum women for early detection and intervention. ^9^ Interventions for patients with anxiety can include both pharmacological and non-pharmacological methods, such as individual or group psychotherapy, music therapy, massage, meditation and hypnotherapy. ^19^^–^^21^

Current recommendations suggest implementing interventions to reduce anxiety before or near the time of cesarean section. ^22^ While some studies using informative videos about spinal block have not shown significant anxiety reduction, ^10^^–^^12^^,^^23^^,^^24^ research utilizing videos specifically about the cesarean operation process itself demonstrated a noteworthy decrease. ^13^ This aligns with our study's finding of reduced post-operative anxiety levels. Interestingly, both the intervention and control groups showed lower anxiety levels post-operatively. This may be attributed to the successful completion of surgery and the absence of complications, leading to a reduction in natural anxiety in both groups. However, a noteworthy detail emerged: the intervention group displayed a higher reduction in the STAI score post-operatively compared to the control group. Furthermore, the results revealed a shift within the intervention group, with 78 percent of patients initially experiencing moderate-to-high anxiety transitioning to a low anxiety level post-operatively, compared to only 34 percent in the control group. This substantial improvement reflects a greater efficacy of the intervention in mitigating presurgical anxiety compared to the control group. Notably, the overall prevalence of moderate anxiety in the intervention group post-operatively was 15 percent, which is consistent with the study of cesarean operative anxiety levels observed in Cindy-Lee's study involving unplanned cesarean operations and natural births. In stark contrast, the control group exhibited a significantly higher prevalence of moderate-to-high anxiety post-operatively, reaching 68 percent. ^18^ All pre-operative intervention patients who received psychological counseling showed no remaining cases of high anxiety level post-surgery, suggesting the effectiveness of counseling in reducing anxiety. However, both groups saw 10 percent (five cases) in the low anxiety group to moderate anxiety. Unfortunately, due to the limited sample size, a definitive analysis of the causes of this shift was not performed.

Previous research has identified several factors that significantly influence antenatal anxiety levels, including low to middle income, low educational attainment, a history of abortions, and the responsibility of caring for other children. Additionally, postnatal concerns, such as breastfeeding difficulties, wound pain, and infant care have been linked to post-operative anxiety. ^9^^,^^16^ Interestingly, our study found that none of the previously identified factors, even a history of abortions, significantly impacted pre-operative anxiety. Instead, watching informative videos before surgery demonstrably reduced anxiety levels post-operatively, consistent with findings from many studies. ^10^^,^^13^^,^^25^

However, a limitation of our study is that it solely assessed anxiety on postpartum day 1 by using the STAI. ^14^ Consequently, factors such as breastfeeding difficulties, wound pain, and infant care challenges, which often arise later in the postnatal period, were not addressed. Previous studies have indicated that postnatal depression can persist for up to 2-3 years. ^6^^,^^7^ Therefore, further research to develop more effective intervention methods and tools aimed at reducing anxiety during pregnancy and motherhood should be considered. Furthermore, our research did not assess the effects of implementing the video intervention 1 week prior to Cesarean delivery; this presents a promising avenue for future investigation.

The findings from this research show that pre-operative video education has emerged as a powerful tool for reducing cesarean operative anxiety, offering promise as a safe, cost-effective, and widely accessible intervention. These findings suggest that informative videos can be disseminated beyond hospital walls, offering benefits to patients who might not otherwise have access to accurate and reliable information. The ability to disseminate and empower patients with clear and accurate pre-operative information regardless of their source of prior knowledge is crucial. In particular, when considering the varied sources from which patients could acquire information pre-operatively, including personal experiences, hearsay, and academic resources. This study highlights the need for comprehensive and reliable pre-operative information to ensure that patients feel empowered throughout their surgical journey.

Furthermore, leveraging technology and digital platforms could expand the reach of educational resources, ensuring that accurate and comprehensive information is easily accessible to a wider audience, creating easily digestible and reliable content that can empower pregnant patients and enable them to make informed decisions and better prepare for surgical procedures. This patient-centered approach not only enhances care and satisfaction but also contributes to improved health outcomes.

## Conclusion

Pre-operative educational videos have emerged as a powerful tool that significantly reduces cesarean operative anxiety and holds promise for improved health outcomes. This readily implementable intervention empowers women with clear and reliable information, transcends hospital walls, and promotes informed decision making. Further research should explore its long-term impact and applicability to other surgical contexts to implement positive surgical experiences. Investing in this approach will not only equip mothers with confidence but also lay the groundwork for a healthier future for mothers and their families.

## Ethics and consent

This randomized controlled trial was approved by the Human Ethics Committee of Thammasat University on the 21 March 2023 (MTU-EC-OB-1-010/66) and Thai Clinical Trials Registry on the 28 March 2023 (TCTR20230328001). Patients who met the selection criteria were reviewed before they were asked to provide written informed consent approved by the ethical approval committee to participate in the project.

## Data Availability

Zenodo: Effect of informative cesarean delivery operative steps video to maternal anxiety level: a randomized controlled trial, https://zenodo.org/records/11372557. ^26^ This project contains following datasets:
1.
Effect of Informative Cesarean Delivery Operative Steps Video to Maternal Anxiety Level.xlsx
2.
information cesarean section (english sub).mp4
3.
Questionnaire.docx Data are available under the terms of the Creative Commons Attribution 4.0 International license (CC-BY 4.0). Zenodo: Effect of informative cesarean delivery operative steps video to maternal anxiety level: a randomized controlled trial, https://zenodo.org/records/11372557. ^26^ This project contains following datasets:
1.
CONSORT-2010-Checklist.doc
2.
Consent Form.docx Data are available under the terms of the Creative Commons Attribution 4.0 International license (CC-BY 4.0).

## Reporting guidelines

Zenodo: CONSORT checklist for ‘Effect of informative cesarean delivery operative steps video to maternal anxiety level: a randomized controlled trial’, https://zenodo.org/records/11372557.

Data are available under the terms of the Creative Commons Attribution 4.0 International license (CC-BY 4.0).
