# Supplementary material for: Effect of Informative Cesarean Delivery Operative Steps Video on Maternal Anxiety Level: A Randomized Controlled Trial
Source: F1000Res. 2024 Dec 23;13:712. Originally published 2024 Jun 28. [Version 3] doi: 10.12688/f1000research.147840.3 (PMC11809628; doi:10.12688/f1000research.147840.3)
Supplement: Peer review history

# Reviewer report: Reviewer response for version 3

**DOI:** 10.5256/f1000research.176027.r351975

**Published:** 2025-01-06

**Authors:** Perapong Inthasorn

**Affiliations:** 1 Faculty of Medicine Siriraj Hospital, Department of Obstetrics & Gynecology, Mahidol University, Bangkok, Thailand

I accept the revision.

Is the work clearly and accurately presented and does it cite the current literature?

Yes

If applicable, is the statistical analysis and its interpretation appropriate?

I cannot comment. A qualified statistician is required.

Are all the source data underlying the results available to ensure full reproducibility?

Yes

Is the study design appropriate and is the work technically sound?

Yes

Are the conclusions drawn adequately supported by the results?

Yes

Are sufficient details of methods and analysis provided to allow replication by others?

Yes

Reviewer Expertise:

NA

I confirm that I have read this submission and believe that I have an appropriate level of expertise to confirm that it is of an acceptable scientific standard.

# Reviewer report: Reviewer response for version 2

**DOI:** 10.5256/f1000research.171685.r324077

**Published:** 2024-09-24

**Authors:** Valentina Lucia La Rosa

**Affiliations:** 1 Department of Educational Sciences, University of Catania, Catania, Italy

I have reviewed the revision of the manuscript entitled "Effect of Informative Cesarean Delivery Operative Steps Video on Maternal Anxiety Level: A Randomized Controlled Trial". The authors have satisfactorily addressed all critical issues and concerns raised in the first revision. The revisions have significantly improved the clarity and quality of the manuscript. The methodology is now well articulated, and the results are more fully presented.

 I believe the manuscript now meets the journal's standards and makes a valuable contribution to the field. I therefore recommend that it be accepted for indexing.

Is the work clearly and accurately presented and does it cite the current literature?

Partly

If applicable, is the statistical analysis and its interpretation appropriate?

Yes

Are all the source data underlying the results available to ensure full reproducibility?

Partly

Is the study design appropriate and is the work technically sound?

Yes

Are the conclusions drawn adequately supported by the results?

Partly

Are sufficient details of methods and analysis provided to allow replication by others?

Partly

Reviewer Expertise:

Developmental and perinatal psychology

I confirm that I have read this submission and believe that I have an appropriate level of expertise to confirm that it is of an acceptable scientific standard.

# Reviewer report: Reviewer response for version 1

**DOI:** 10.5256/f1000research.162078.r297337

**Published:** 2024-12-13

**Authors:** Perapong Inthasorn

**Affiliations:** 1 Faculty of Medicine Siriraj Hospital, Department of Obstetrics & Gynecology, Mahidol University, Bangkok, Thailand

This is randomized controlled trial compared anxiety level in planned cesarean pregnant women between intervention group(informative VDO watching ) and control group. The study showed that STAI score of the intervention and control groups did not differ significantly before cesarean delivery. However the postoperative score had significantly decrease in intervention group compared to control group.

 I would like the authors to discuss about this topics.

 1. Why anxiety did not decrease preoperatively?

 2.In intervention group, could the anxiety decrease preoperatively, If the subjects in intervention group watched VDO 1 week before cesarean section ?

Is the work clearly and accurately presented and does it cite the current literature?

Yes

If applicable, is the statistical analysis and its interpretation appropriate?

I cannot comment. A qualified statistician is required.

Are all the source data underlying the results available to ensure full reproducibility?

Yes

Is the study design appropriate and is the work technically sound?

Yes

Are the conclusions drawn adequately supported by the results?

Yes

Are sufficient details of methods and analysis provided to allow replication by others?

Yes

Reviewer Expertise:

I am obstetrician and gynaecologist specialized in gynaecologic oncologist.

I confirm that I have read this submission and believe that I have an appropriate level of expertise to confirm that it is of an acceptable scientific standard, however I have significant reservations, as outlined above.

# Response

**Published:** 2024-12-15

**Authors:** Panicha Phetprapasri

**Affiliations:** Obstetrics and Gynecology, Thammasat University, -, Pathum Thani, Thailand

I have carefully reviewed the feedback and have made the required revisions in the "Submit Version 3" section of F1000 Research. Thank you for your guidance and consideration.

# Reviewer report: Reviewer response for version 1

**DOI:** 10.5256/f1000research.162078.r311443

**Published:** 2024-08-21

**Authors:** Valentina Lucia La Rosa

**Affiliations:** 1 Department of Educational Sciences, University of Catania, Catania, Italy

The manuscript “Effect of Informative Cesarean Delivery Operative Steps Video on Maternal Anxiety Level: A Randomized Controlled Trial” provides valuable insights into a practical intervention aimed at reducing maternal anxiety during cesarean delivery. The study is well conducted and presents its findings clearly, but there are several areas where additional detail and discussion are needed to fully contextualize the findings. Below are some comments and suggestions for improving the manuscript:

**Major Revisions**

1. In the Introduction, I recommend expanding the discussion of the effects of cesarean delivery on maternal mental health, particularly highlighting the differences between planned (elective) cesarean sections and emergency cesarean sections. The psychological effects, including anxiety and postpartum depression, may differ significantly between these two types of procedures and should be further explored. Including a more detailed review of relevant studies [ref 1,2 and 3] would strengthen the background of your study.
2. The study exclusion criteria appear to be comprehensive, but may potentially limit generalizability. In particular, the exclusion of women with pre-pregnancy mental disorders may introduce selection bias. These women may represent a significant proportion of those who experience anxiety prior to cesarean section. I recommend expanding the discussion of how this limitation affects the generalizability of the study findings.
3. The STAI was only used to assess anxiety up to postpartum day 1. Anxiety may manifest or change in the later postpartum period due to additional factors such as breastfeeding difficulties or infant care. Consider discussing the rationale for this limited follow-up period and recognize the need for longer-term assessment of maternal anxiety in future research.
4. The authors should provide more detailed information on the psychometric properties of the State-Trait Anxiety Inventory. Specifically, it would be beneficial to include data on its reliability (e.g., Cronbach's alpha) and validity within the study sample.
5. Although the manuscript provides a detailed description of the video, more information is needed about its content development. Was the video content tested for comprehension and appropriateness before being used in this study? It would be helpful to include details on how the video was validated or pre-tested for clarity and accuracy.
6. Although the study enrolled 178 participants, a sample size calculation or power analysis to justify the number of participants is not clearly provided in the text. Please clarify if this was done and include it in the methods section.
7. The discussion is thorough, but lacks a deeper exploration of how the findings are consistent with or contradict existing literature. The authors should expand on how their findings compare to other studies that have examined similar interventions, particularly those with different outcomes. Discussion of possible mechanisms for the observed reductions in anxiety would strengthen the paper's contribution to the field.
8. The manuscript mentions ethical approval, but it would benefit from a more detailed discussion of how informed consent was obtained, particularly with regard to how participants were informed about the randomization process and the potential benefits/risks of watching the video.

**Minor Revisions**

1. The manuscript is generally clear, but there are a few areas where language could be improved for clarity. For instance, some sentences in the discussion section are overly complex. Consider simplifying them to improve readability.

Is the work clearly and accurately presented and does it cite the current literature?

Partly

If applicable, is the statistical analysis and its interpretation appropriate?

Yes

Are all the source data underlying the results available to ensure full reproducibility?

Partly

Is the study design appropriate and is the work technically sound?

Yes

Are the conclusions drawn adequately supported by the results?

Partly

Are sufficient details of methods and analysis provided to allow replication by others?

Partly

Reviewer Expertise:

Developmental and perinatal psychology

I confirm that I have read this submission and believe that I have an appropriate level of expertise to confirm that it is of an acceptable scientific standard, however I have significant reservations, as outlined above.

## References

[1] : Trait anxiety and unplanned delivery mode enhance the risk for childbirth-related post-traumatic stress disorder symptoms in women with and without risk of preterm birth: A multi sample path analysis. *PLoS One* .2021;16(8) : 10.1371/journal.pone.0256681 e0256681 10.1371/journal.pone.0256681 34464408 PMC8407573

[2] : Giving birth alone due to COVID-19-related hospital restrictions compared to accompanied birth: psychological distress in women with caesarean section or vaginal birth - a cross-sectional study. *J Perinat Med* .2022;50(5) : 10.1515/jpm-2021-0368 539-548 10.1515/jpm-2021-0368 35357796

[3] : The association between caesarean and postnatal psychological distress: Effect modification by mental health history. *Paediatr Perinat Epidemiol* .2021;35(6) : 10.1111/ppe.12791 635-644 10.1111/ppe.12791 34255373

# Response

**Published:** 2024-09-09

**Authors:** Panicha Phetprapasri

**Affiliations:** Obstetrics and Gynecology, Thammasat University, -, Pathum Thani, Thailand

I have carefully reviewed the feedback and have made the required revisions in the "Submit New Version" section of F1000 Research. Thank you for your guidance and consideration.
